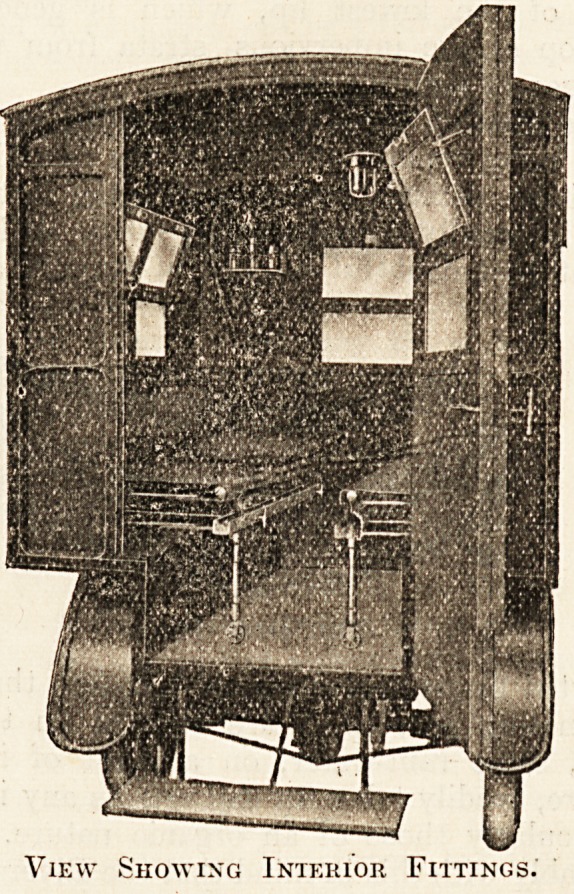# A New Type of Motor Ambulance

**Published:** 1911-02-25

**Authors:** 


### A NEW TYPE OF MOTOR AMBULANCE.

One of the most marked signs of progress in civil and municipal life is exemplified in a comparison between the old methods of handling the injured and the facilities that are now available for the careful and prompt conveyance of victims of accident or disaster to doctor, hospital, or infirmary. Only a decade ago any conveyance was regarded as suitable to carry an injured person, while the ambulance proper—presumably built for the specific purpose—was a ramshackle vehicle, often devoid of any but the most rudimentary type of springs, and from a "comfort" point of view was little better than a farm waggon. A few years have sufficed to alter this system out of recognition, and the efforts of our big cities and municipal bodies have demonstrated once more the all-conquering superiority and advantages of the motor-ambulance for the prompt transference of injured to a place where the necessary attention can be given. We append a few comparisons which, without technicalities, will probably be of special interest, and it may be added that a much larger number of medical officers, heads of hospitals, constabularies, mining officials, aldermen, and councillors are vastly interested in this important subject than the general public would at first thought imagine :—

| Old Type. | Present Day. |
|---|---|
| *Speed a most Important Point.* | |
| At the best, a jog trot, often reduced to a walk, on account of the patient's suffering. Many an accident which might have been treated successfully has terminated fatally owing to the length of time occupied on the journey. | Anything up to 12 miles per hour by modern Horsed Ambulance, or up to 40 miles per hour by Motor Ambulance. |
| *Comfort of and Attention to Patients.* | |
| In the old ambulance the patient was placed upon a rude litter, which rested *upon the floor* of the vehicle, and was subject to all the violent oscillation and vibration set up by roughness of road, wheels with iron tyres, and the rigid, inelastic nature of the conveyance. | Nowadays the motor or horsed ambulance has rubber tyres and the most elastic and resilient spring suspension, supplemented in the motor ambulance with pneumatic tyres if necessary. The stretcher itself upon which the patient is laid (well above the floor) is of an elastic canvas foundation, and this stretcher in turn is mounted in some cases on special patent suspension springs, effectually absorbing any vibration which might be set up. Surgical requisites, oxygen, stimulants and first aid appliances are always available. |
| *Interchangeability.* | |
| The old ambulance was regarded as of no utility for any purpose except that for which it was purchased, i.e., the conveyance of a single patient or injured person. | The present car, horsed or motor, is invariably fitted with a cushioned seat, for passengers or convalescents; in practically all cases accommodation is provided for *two* injured persons, and in many instances both stretchers are removable. |

Our photographs illustrate the progressive policy adopted by the Fire Department of Pretoria, in purchasing what may be regarded as the most complete and comprehensive vehicle of this character which has ever been conceived, and we are indebted to the builders, Messrs. J. & A. Carter, of 2, 4, and 6 New Cavendish Street, W., for the following particulars :—

The chassis is of 1911 pattern, fitted with "Silent Knight" four-cylinder engine; four speeds, specially geared for ambulance work and hill climbing, with a Colonial clearance of 9 inches from the ground. Solid tyres are fitted to this model for traversing rough roads in country districts; three brakes. The body of the car affords

Pretoria Motor-ambulance : Exterior.

unique accommodation; the stretchers along each seat-line are drawn towards the centre door at the rear, and the loading of stretchers is facilitated by an extension piece upon which the stretcher can rest, and this obviates the necessity for attendant climbing into the car, a difficult and dangerous operation when carrying an injured person. The stretchers and spring frames upon which they rest are

View Showing Interior Fittings.

collapsible, folding into small compass for packing, and both are easily detachable, so that the car can be instantly a travelling conveyance for passengers or convalescents, the spring stuffed cushioned seats and back-rests, combined with the special resilience of the springs, eliminating all jar or vibration. The car is designed for a maximum load of thirteen persons.

## Figures and Tables

**Figure f1:**
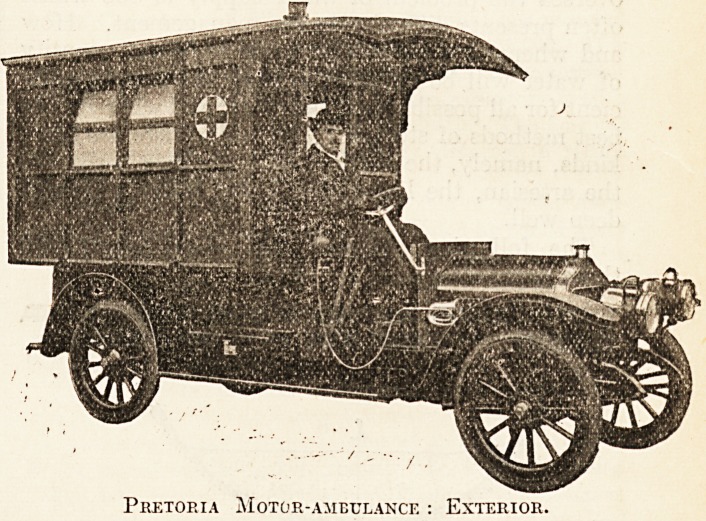


**Figure f2:**